# Profile: Dr Bourne’s identity - credit where credit’s due

**DOI:** 10.1192/pb.bp.113.046060

**Published:** 2014-04

**Authors:** Jonathan Pimm

To be responsible for saving hundreds if not thousands of lives is no mean feat. It ought to bring appreciation and recognition. However, for Dr Harold Bourne, a man whose actions prevented innumerable deaths and misery, this was not the case.

The reasons why such a state of affairs came to pass are tied up in the story of an individual’s fight for justice with no fear of the consequences: one man taking on the mainstream medical profession in order to stop use of a treatment that had been harming and killing people across the world for more than a quarter of a century. This classic David and Goliath battle began 60 years ago, when Bourne decided to blow the whistle on a well-established treatment for schizophrenia, insulin coma therapy.

Unfortunately for Bourne, one of the major protagonists of the treatment was Dr William Sargant, an extremely powerful and highly regarded director of a leading psychiatric unit at a London medical school. Sargant and a Dr Eliot Slater - another big gun in the specialty at the time - were authors of a major textbook advocating the use of physical treatments for mental disorders, including insulin coma therapy, psychosurgery and electroconvulsive therapy (ECT).^1^

Armed with only a razor-sharp intellect and a desire to follow the dictum of non-maleficence, Bourne first raised his concerns in a paper published in *The Lancet* in 1953.^2^ Interestingly, he had submitted the article to the *Journal of Mental Science* (later to become the *British Journal of Psychiatry*) a year earlier but it had been rejected; having had no reply after 12 months, Bourne inquired what had become of his work and was told it could not be published and he should get ‘more experience’ (H. Bourne, personal communication, 2013). Entitled simply ‘The insulin myth’, the paper showed there to be no basis for the belief that the radical treatment, involving putting patients into a deep coma for up to 15 mins five or six mornings a week for as much as 10 weeks, had a beneficial effect in the treatment of schizophrenia. Such a dismissal of the well-established therapy was utterly shocking at the time and for it to have been made by such a young, inexperienced doctor really rubbed salt in the wounds of the scientific elite.

Insulin coma therapy had been developed by Dr Manfred Sakel at the University Neuropsychiatric Clinic in Vienna. In the late 1920s, Sakel found that small doses of insulin helped morphine addicts with their withdrawal symptoms. And when he tried giving it to psychotic patients he noted improvement. His work attracted international attention and doctors from many countries all over the world came to study the treatment. As described in several textbooks - including that by Sargant & Slater^1^ - the treatment (known as deep insulin coma therapy or DICT) was extremely rigorous. It was administered in separate units with the patients staying together with the same doctors and nurses throughout. Comas were induced with doses of typically 10-15 units of insulin, which made patients hypotonic without corneal or papillary reflexes. The hypoglycaemia made patients very restless and liable to major convulsions.

The treatment was very labour intensive, with patients requiring continuous nursing supervision for many hours after the actual coma because they were liable to further hypoglycaemic ‘after shocks’.^3^ There was a mortality rate of about 1% as well as a liability to permanent brain damage, although figures on the number of deaths and the morbidity associated with the treatment are difficult to obtain. However, it is highly likely that, because of the upbeat enthusiasm with which the therapy - and all physical treatments for that matter - was embraced, any negative headlines were brushed aside and largely ignored.

Indeed, insulin coma therapy was adopted by the establishment very quickly and by 1938 it was being used extensively in 31 hospitals in England and Wales. Interestingly, European doctors, especially those fleeing Nazism, were employed to introduce it. A leading hands-on insulin therapist was Dr Wilhelm Mayer-Gross, who had a most distinguished career in Germany before escaping to the UK. He shared with Slater and Sir Martin Roth the authorship of *Clinical Psychiatry*,^4^ another standard textbook of the time. The book advocated insulin coma therapy and referred to Mayer-Gross’s extensive (10 years’) experience with the treatment in an attempt to validate its continued use. Indeed, the 1960 edition still advocated the use of the therapy for patients with schizophrenia 7 years after the insulin myth paper and 2 years after the ‘Insulin coma in decline’ paper (also by Bourne).^2,5^ Bourne’s article sparked many leading psychiatrists to send condemnatory criticisms to *The Lancet*. Their tone was typified by remarks such as ‘it is clinical experience that counts here, despite all figures to the contrary’.^3^

With the publication of another randomised controlled trial in *The Lancet* showing that insulin was not useful in the treatment of schizophrenia, the tide of concern about the treatment was rising fast.^6^ Fired up by this evidence, Bourne again released a slingshot attack on the giants in the establishment.^5^ But this time he published his blistering dismissal of the treatment in an American journal and from a position of relative safety as a lecturer in psychiatry at the University of Otago, in Dunedin, New Zealand. Bourne had moved to the Antipodes for several reasons but mainly because he had been unable to secure himself a decent job in England on account of the bad feelings that had developed towards him from senior psychiatric colleagues.

The professional eminence of the main advocates of the use of insulin coma therapy is worth reiterating since it undoubtedly led to the diminishment of the academic and professional rewards that should have been heaped upon Bourne. Further, such prolonged use and advocacy of an ineffective treatment by pillars of the psychiatric profession meant that even though insulin coma therapy had largely fallen out of use in Britain and the USA by the 1970s, it was still being practised and researched in some hospitals^7^ and may have continued for longer in countries such as China and the Soviet Union.^8^ And even more bizarrely, the leading advocates of the treatment suffered no repercussions as far as their own careers were concerned. Specifically, Sargant became president of the section of psychiatry at the Royal Society of Medicine and a founding member of the World Psychiatric Association. He was awarded the Starkey medal and prize by the Royal Society of Health for his work on mental health. He also had a large private practice in London’s Harley Street. He wrote articles for the medical and popular press, appeared in TV programmes and published an autobiography, *The Unquiet Mind*, in 1967. He died in 1988.

As for Slater, he was made editor of the *British Journal of Psychiatry* from 1961 to 1972. And in 1966 he was awarded a CBE. He also held honorary fellowships of several British, German and American medical and psychiatric societies, as well as of his Cambridge College, St John’s. In 1971, he received an honorary degree from the University of Dundee. He died in 1983.

**Fig 1:**
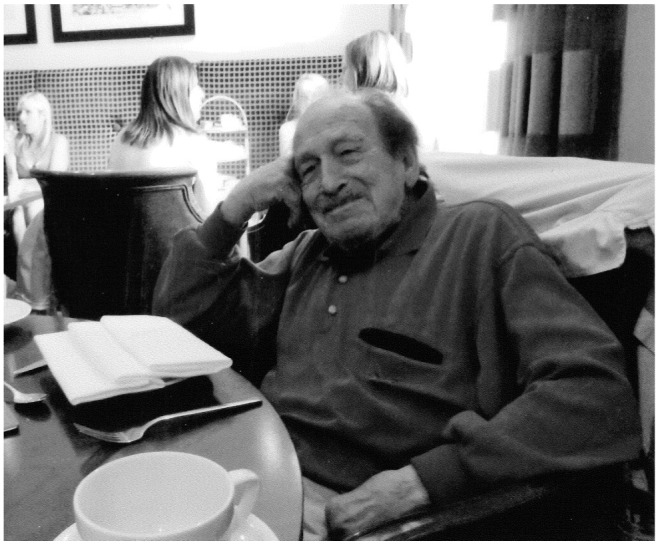
Dr Harold Bourne taking supper in Rome.

But what about Bourne? He has received some recognition for his work in the form of the Evan Jones Prize for the ‘most distinguished contribution to psychiatry in Australasia’. He is 90 years old and lives in a flat in the rather romantic-sounding Via Pietro de Cristofaro, about a 20-minute walk east of the Vatican City area of Rome. He is married to an Italian psychoanalytical psychiatrist with whom he has two grown up children; she lives across the road from him in another flat. The precise reasons for this arrangement are not clear even to Bourne. He continues to see a few patients for therapy. And has up until relatively recently continued to publish a steady stream of articles and letters in the academic press.^9,10^ He rises every morning and still manages to get to his local bar for a cappuccino and to read *Il Messagero* (Fig. 1).

He was born and brought up in Tottenham in north London and won a scholarship to the local school; modestly he explains that he was the only one who applied for it so the odds were very much in his favour. ‘In 1938 I discovered Freud and Marxism. I read Freud and Marx in my early teens and by late teens I decided then that I wanted to become a psychoanalyst’, he said. ‘In my teens I found myself being someone who was always against the government, I was a Marxist and a member of the British Communist Party,’ he added.

He was later expelled from the Communist Party. He said: ‘I have met lots of people who joined the party and left of their own accord - but I never met anyone else who had been expelled’.

He studied medicine at University College Hospital in London and graduated in 1945. At the age of 23 years, he was married with two young children and started working in a military hospital. The job came to an end in 1950 and Dr Bourne decided that he should pursue his ambition to train in psychoanalysis. However, because he could not afford to pay for his own therapy as part of the training, he determined to focus on obtaining the necessary examinations. He then published the landmark insulin paper, after which he ‘became unemployable in any self-respecting psychiatric department’. He briefly took a position at a hospital for patients with intellectual disability but then moved to New Zealand. After 18 years in Dunedin at the Otago Medical School, Dr Bourne decided to return to England. He said: ‘I really needed psychoanalysis because I had got into fearsome troubles with my wife... I think analysis is essential as a means of studying the workings of the human mind’.

He obtained a position at Banstead Hospital where he developed a therapeutic community which, he says, ‘had visitors from all over the world’. It was also around this time that he met and married his second wife. A few years later, in 1989, when he retired from the National Health Service at the age of 66, he moved to Italy.

During an interview with the *Psychiatric Bulletin* in his flat in Rome, Bourne admitted that he feels ‘kind of irritated’ about how things turned out after the iconoclastic insulin paper was published. Essentially, Bourne’s desire to see that the widely used treatment was at least backed up by some form of evidence base was the catalyst that set the track for his whole career. However, I, for one, believe that Bourne’s courage and determination to stick to his principles and not to swim with the tide of professional opinion should be praised and admired. And he should look back on such an outstanding act of individual heroism (along with all his other rather remarkable professional achievements) with joy, satisfaction, contentment and pride. Such emotions are priceless.
